# Fetal Heart Rate Variability Is Affected by Fetal Movements: A Systematic Review

**DOI:** 10.3389/fphys.2020.578898

**Published:** 2020-09-30

**Authors:** Anne Rahbek Zizzo, Ida Kirkegaard, John Hansen, Niels Uldbjerg, Henning Mølgaard

**Affiliations:** ^1^Department of Obstetrics and Gynecology, Aarhus University Hospital, Aarhus, Denmark; ^2^Department of Health Science and Technology, Aalborg University, Aalborg, Denmark; ^3^Department of Cardiology, Aarhus University Hospital, Aarhus, Denmark

**Keywords:** electrocardiagram, autonomic nerous function, heart rate variabiity, sinus arrhythmia, fetus (MeSH), pregnanant women

## Abstract

**Introduction:** Fetal heart rate variability (FHRV) evaluates the fetal neurological state, which is poorly assessed by conventional prenatal surveillance including cardiotocography (CTG). Accurate FHRV on a beat-to-beat basis, assessed by time domain and spectral domain analyses, has shown promising results in the scope of fetal surveillance. However, accepted standards for these techniques are lacking, and the influence of fetal breathing movements and gross movements may be especially challenging. Thus, current standards for equivalent assessments in adults prescribe rest and controlled respiration. The aim of this review is to clarify the importance of fetal movements on FHRV.

**Methods:** A systematic review in accordance with the PRISMA guidelines based on publications in the EMBASE, the MEDLINE, and the Cochrane Library databases was performed. Studies describing the impact of fetal movements on time domain, spectral domain and entropy analyses in healthy human fetuses were reviewed. Only studies based on fetal electrocardiography or fetal magnetocardiography were included. PROSPERO registration number: CRD42018068806.

**Results:** In total, 14 observational studies were included. Fetal movement detection, signal processing, length, and selection of appropriate time series varied across studies. Despite these divergences, all studies showed an increase in overall FHRV in the moving fetus compared to the resting fetus. Especially short-term, vagal mediated indexes showed an increase during fetal breathing movements including an increase in Root Mean Square of the Successive Differences (RMSSD) and High Frequency power (HF) and a decrease in Low Frequency power/High Frequency power (LF/HF). These findings were present even in analyses restricted to one specific fetal behavioral state defined by Nijhuis. On the other hand, fetal body movements seemed to increase parameters supposed to represent the sympathetic response [LF and Standard Deviation of RR-intervals from normal sinus beats (SDNN)] proportionally more than parameters representing the parasympathetic response (RMSSD, HF). Results regarding entropy analyses were inconclusive.

**Conclusion:** Time domain analyses as well as spectral domain analyses are affected by fetal movements. Fetal movements and especially breathing movements should be considered in these analyses of FHRV.

## Introduction

Low fetal heart rate variability (FHRV), as assessed by conventional cardiotocography (CTG), is associated with fetal acidosis and even fetal death (Hon and Lee, 1963), but also with a relatively poor clinical performance (Alfirevic et al., 2017). In the context of FHRV, one major limitation of conventional CTG is that it does not provide the opportunity to perform variability analyses on a beat-to-beat level due to both the complex doppler signal corresponding to a single beat and the averaging of inter beat intervals (Peters et al., 2001). In beat-to-beat time domain and spectral domain analyses, a fixed marker within the heartbeat signal (fiducial point) and a high sampling frequency are required. Magnetocardiography (MCG) and electrocardiography (ECG) meet these requirements. Fetal MCG (FMCG) is based on a relatively complicated set up applicable during pregnancy but hardly during active labor. During labor, Fetal ECG (FECG) is obtainable by scalp electrode, and during pregnancy, albeit with challenges, by electrodes placed at the maternal abdomen non-invasive fetal electrocardiography (NI-FECG). At present MCG is primarily used in scientific setups, while NI-FECG is implemented in clinical setups for obtaining CTG. NI-FECG is easy to use and has therefore a potential in home monitoring settings.

Respiratory sinus arrhythmia (RSA) is a well-known physiological rhythm in adults, neonates, and fetuses caused by primarily respiratory modulated variations in efferent vagal activity, and its amplitude and frequency depends on the respiratory frequency. HRV (measured as variability of RR-intervals in milliseconds) including RSA, is often assessed by time domain and spectral domain analyses and relates to the prognosis of various conditions. High values relate to a healthy and good prognosis (Heart rate variability, 1996). Studies on newborns have demonstrated the potential of such HRV analyses, as they constitute an extremely early biomarker of neonatal sepsis (Griffin and Moorman, 2001) and are strongly associated with the development of hypoxic ischemic encephalopathy (Vergales et al., 2014) and outcome after respiratory distress syndrome (Nishida et al., 1981). Studies in fetuses suggest that FHRV analysis may predict fetal distress during labor (Van Laar et al., 2008) and mental and psychomotor development at 2 years (DiPietro et al., 2007). In addition, several studies have shown a correlation between FHRV and fetal growth restriction (FGR) (Nijhuis et al., 2000; Kikuchi et al., 2006; Fukushima et al., 2011; Goncalves et al., 2013; Arias-Ortega et al., 2016).

Proper acquisition of NI-FECG at high sampling rates has become technically possible. Therefore, it is time to establish methodological standards concerning factors affecting FHRV analyses, to provide meaningful interpretation and comparison of results and consistency for future studies. Potential variables of importance include gestational age (Van Leeuwen et al., 2003, 2013; Schneider et al., 2018), diurnal rhythm (Morokuma et al., 2001; Kapaya et al., 2018), fetal breathing movements (Timor-Tritsch et al., 1977), fetal gross movements (van Laar et al., 2014), fetal behavioral states (Goncalves et al., 2007), smoking (Kapaya et al., 2015; Spyridou et al., 2017), fetal gender (Bernardes et al., 2008; Tendais et al., 2015; Goncalves et al., 2017), ethnic differences (Marie et al., 2015; Tagliaferri et al., 2017), and maternal exercise (May et al., 2010). Fetal movements of the extremities and trunk, as well as fetal breathing movements, are intermittent and unpredictable; thus, it is especially important to know their influence on the results of FHRV analyses. It may be important to assess fetal movements in a way which is both feasible and reliable; potential methods include (1) fetal activity reported by the mother, however with low sensitivity (Sorokin et al., 1982; Schmidt et al., 1984); (2) accelerations and variability at CTG, also with low sensitivity at early gestational age (Pillai and James, 1990a); (3) real-time detection by ultrasound (Groome et al., 1994); (4) myography (Ulusar et al., 2011); and (5) actocardiography (Brändle et al., 2015). Detection of fetal movements is often classified into Specific Movement Pattern (SMPs), which involves specification of the movement of a certain body part (head, arm, leg etc.) and Non-Specific Movement Pattern (NSMPs) including terms like activity, body activity, gross movements, and trunk movements (de Vries et al., 1982, 1985). Additionally, fetal behavioral states (FBS) defined by Nijhuis et al. (1982) are also used in the differentiation of an active and a resting fetus. In this review fetal movements are divided into two categories: (1) breathing vs. non-breathing and (2) body movements (activity/gross movements/movements of extremities/active stages of FBS after 35 weeks) vs. rest.

The aim of this review is to clarify whether FHRV measurements in the form of time domain and spectral domain parameters should consider fetal movements including fetal breathing movements.

## Methods

### Protocol and Registration

The systematic review was conducted in accordance with the PRISMA (Preferred Reporting Items for Systematic Reviews and Meta-Analyses) statement (Moher et al., 2009). A predefined review protocol was performed and can be accessed through PROSPERO (registration number: CRD42018068806). https://www.crd.york.ac.uk/PROSPERO/display_record.php?RecordID=68806.

### Outcome Measures

Time domain, spectral domain and entropy parameters, and the association with (1) breathing vs. non-breathing and (2) body movements (activity/gross movements/movements of extremities/active stages of FBS after 35 weeks) vs. rest.

Examples of time domain parameters are standard deviation of normal-to-normal (NN) interval (SDNN), where normal-to-normal refers to intervals between QRS-complexes arising from normal sinus beats, and root mean square of the successive differences (RMSSD). Spectral parameters reflects the frequency of “the melodies” in the variance of NN-intervals and include low frequency power (LF-power), high frequency power (HF-power) and the ratio (LF/HF-power). Entropy analyses are techniques used to quantify the amount of repetitive patterns and unpredictability in NN-intervals, examples are approximate entropy (ApEn) and sample entropy (SampEn). Ref. task force.

### Data Sources

Studies assessing FHRV in relation to fetal movements were searched systematically through EMBASE, MEDLINE, and Cochrane online databases. The last literature search was performed on 30th of January 2020. The literature search was based on the research PICOS (Appendix 1). Search terms are listed in Table 1.

**Table 1:** Search strategy and criteria for the selection of eligible studies.

**Search strategy**	
Search terms (including their synonyms and MeSH terms) were combined according to PICO:	
	“fetus”, “fetal”, “pregnancy” AND “active”, “respiration”, “fetal movement”, “fetal behavioral state”, “sinus arrhythmia”, AND “entropy”, “heart rate variability”, “linear models”, “nonlinear dynamics”, “spectral”, “time domain”, “short-term variation”, “short-term variability”, “frequency analysis”, “fractals”, “magnetocardiography”, “electrocardiography”, “electrocardiogram”, “magnetocardiogram”
Eligibility criteria	
Inclusion criteria	
	(1) Singleton uncomplicated pregnancies, before start of labor. (2) Fetal heart rate detection based on Electrocardiography (ECG) or Magnetocardiography (MCG). (3) Reporting on both moving and resting fetuses. (4) Time domain Standard Deviation of normal-to-normal intervals (SDNN), Root Mean Square of the Successive Differences (RMSSD), Spectral (Very low Frequency power (VLF), Low Frequency Power (LF), High Frequency power (HF), LF/HF, total power) and Entropy [Approximate Entropy (ApEN), Sample Entropy (SampEn)] analyses
Exclusion criteria	
	(1) Complicated pregnancies (2) Compromised fetuses (3) Fetal activity based only on Maternal Perception or Heart Rate Pattern (HRP) before 35 gestational weeks
Time frame	
	All years
Language	
	English
Publication status	
	Peer reviewed articles

	No animal studies

### Eligibility Criteria

Only studies on singleton pregnancies describing FHRV in fetuses moving compared to fetuses not moving were considered for inclusion. In addition, included studies had to report either time domain, spectral or entropy (ApEn, SampEn) analyses based on FECG or FMCG. Complicated pregnancies or compromised fetuses were excluded to avoid possible confounders including fetal growth restriction (FGR) and gestational diabetes mellitus (GDM). If the study reported on both normal and complicated pregnancies, the normal pregnancies of these studies were included (Gustafson et al., 2012; Arias-Ortega et al., 2016). Studies defining fetal activity on maternal perception in all pregnancies or heart rate pattern (HRP) before 35 gestational weeks were also excluded.

### Data Extraction and Quality Assessment

**Figure 1:**
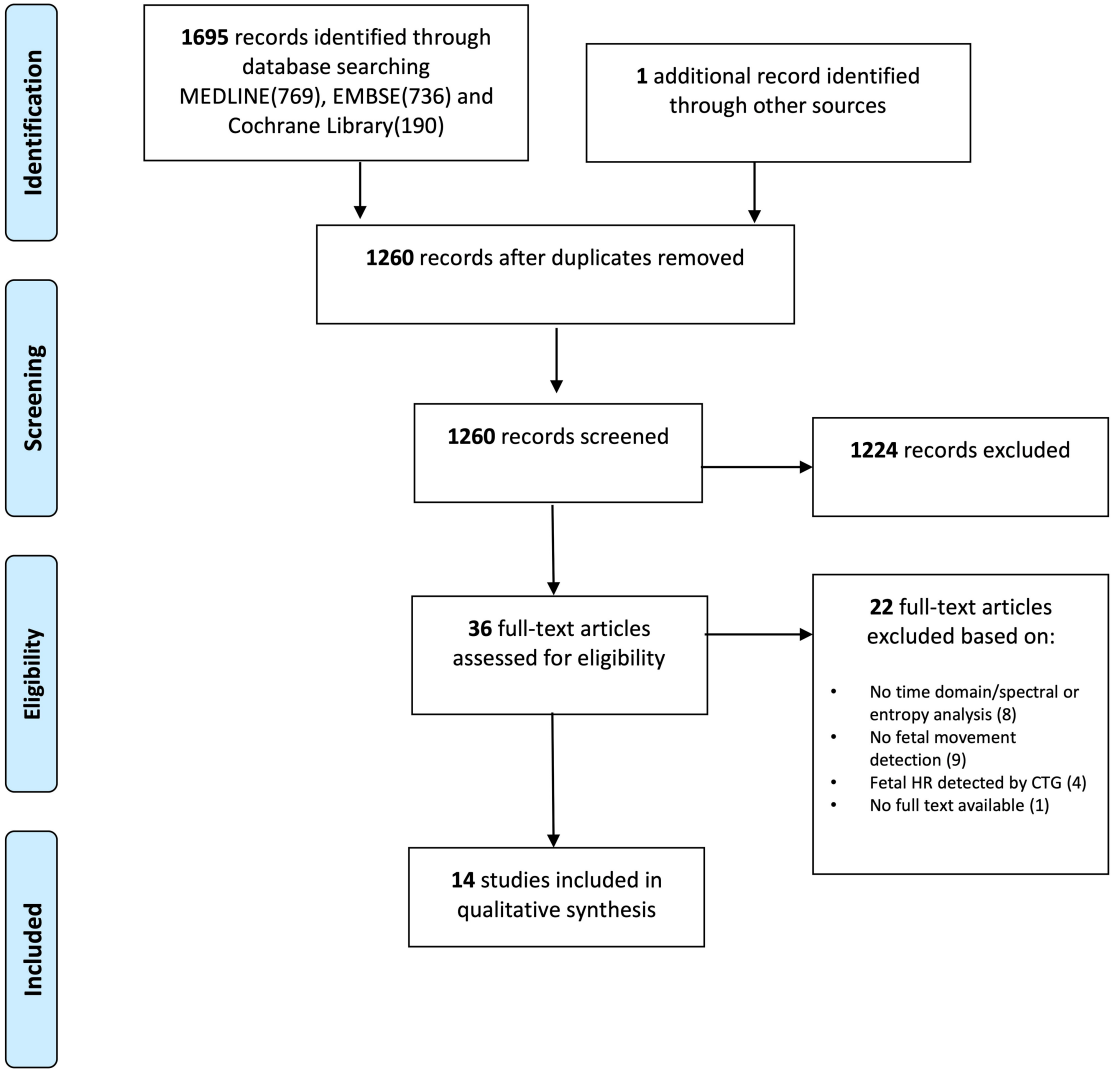
PRISMA flow diagram showing identified, included, and excluded studies.

Two authors (AZ and IK) selected relevant publications (Figure 1). They independently screened titles and abstracts, crosschecked reference lists for further relevant papers, and conducted full-text screening based on the eligibility criteria as described above. In case of disagreements, a third author was consulted to achieve consensus.

Data were extracted from included studies by at least two authors (AZ, IK, HM, NU, JH). Two authors (AZ and NU) conducted the quality assessment. As no suitable quality assessment tool was found, we elaborated our own (Table 2) with the following main topics: risk of allocation, selection, information, detection and reporting bias including design, population, method of data acquisition, method of signal processing and R-wave detection, method of fetal movement detection and heart rate variability analyses.

**Table 2:**
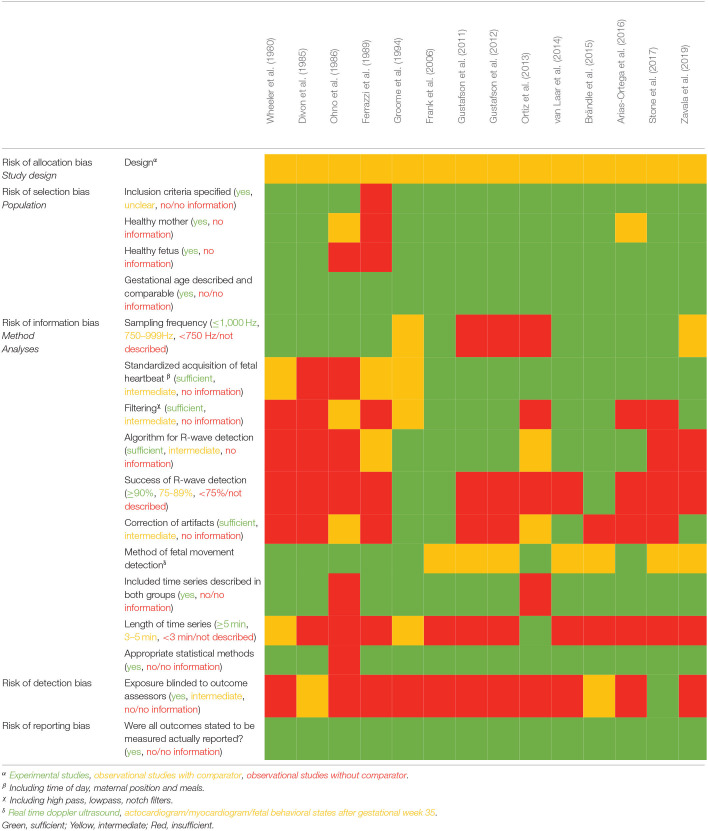
Methodological quality and risk of bias assessment of the included studies.

## Results

The literature search identified 1,260 potentially relevant records, of which 1,224 were excluded based on title and abstract. Therefore, 36 records were assessed for eligibility by full-text assessment, and 14 studies fulfilled the inclusion criteria (Figure 1). No randomized controlled trials were included, which was expected according to the research question. Nine (DiPietro et al., 2007) studies reported on fetal breathing movements and six (Vergales et al., 2014) on fetal body movements/active fetal state. In two (Alfirevic et al., 2017) studies, only a subpopulation met the inclusion criteria, which was used for this review.

Some heterogeneity was seen across studies in the form of various methods of fetal movement detection, definitions of fetal movements, and length of analyzed timeseries; in spectral analyses, slightly different frequency bands were used (Table 3). Most studies included pregnancies around term, but two (Alfirevic et al., 2017) studies only included preterm pregnancies around 34 weeks of gestation (Ortiz et al., 2013; Arias-Ortega et al., 2016), and one (Hon and Lee, 1963) study included pregnancies from 24 to 41 week of gestation (Brändle et al., 2015).

**Table 3:** Included studies, characteristics and outcome.

**Population**				**Method**					**Outcomes**
**References**	**N_**fetus**_/N_**record**_**	**GA in weeks**	**Hz**	**Mode of fetal movement**	**Fetal movement detection**	**Analysis**	**Length of time series**	**Measure/unit**	**Short description**
Wheeler et al. (1980)	21/34	36–41	10,000	Breathing/non-breathing Active/rest	UL	Time domain	Unclear /1-min epochs	RR-interval/ms	SDNN was higher in breathing and active episodes compared to non-breathing and rest. No change in mean RR.
Divon et al. (1985)	15/60	38–41	?	Breathing/non-breathing	UL	Spectral	Unclear/1-min epochs	RR-interval/spectral density	A density peak in 0.7–0.95 Hz during breathing. Frequency range identical with breathing frequency. Spectral densities slightly elevated between 0.7 and 1.05 Hz during non-breathing.
Ohno et al. (1986)	4/32	36–41	?	Breathing/non-breathing	UL	Time domain	256 beat epochs	RR-interval/ms	SDNN was higher in breathing compared to non-breathing. The percentage of beat-to-beat differences below 2 ms, was lower during breathing. No difference in mean heart rate.
Ferrazzi et al. (1989)	4/4	26/36	1,024	Breathing/non-breathing	UL	Spectral	256 beat epochs	RR-interval/s	A HF peak around 0.7–0.9 Hz was seen during breathing in GA 36, not in GA 26.
Groome et al. (1994)	13/81	36–40	833	Breathing/non-breathing	UL	Spectral	3 min	RR-interval/no units	Total power, VLF, LF and HF power were higher in epochs of breathing compared to non-breathing in the quiet FBS (1F). 85% had a prominent HF peak during breathing and 54% in non-breathing, both in quiet FBS (1F)
Frank et al. (2006)	39/39	35–40	1,000	Active/rest	FBS	Time domain Spectral Entropy	5 min	RR-intervals/ms	Active FBS (2F, 4F) was associated with higher variability compared to the less active state (1F) including higher SDNN, RMSSD, ln(LF), ln(HF). ApEn_sub_ decreased with fetal activity (1F vs. 4F) While ApEn_pop_ increased from 1F to 2F. however, unchanged from 1F to 4F.
Gustafson et al. (2011)	?/43	36–38	300	Breathing/non-breathing	dMMG/FBS	Time domain Spectral	Varied from 60 to 144 s	RR-interval/ms	RMSSD and HF were higher in breathing epochs compared to non-breathing epochs in active FBS (2F, 4F). LF/HF and heart rate were lower in breathing epochs compared to non-breathing
Ortiz et al. (2013)	12/26	34 (±3.7)	500	≤ 40% breathing/ <40% non-breathing	UL	Time domain Spectral	5 min	RR-interval/ms	HF and RMSSD were higher during breathing. Both RMSSD and HF, in the group with <40% breathing, showed a significant linear correlation with RR intervals. Both increasing with increasing RR-interval. No significant linear correlation in the group with breathing ≥40 % No difference in Mean RR in the two groups.
Gustafson et al. (2012)	15/15	>36	300	Breathing/non-breathing	dMMG/FBS	Time domain Spectral	Varied from 60 to 140 s	RR-intervals/ms	Fetal breathing resulted in significantly higher Log (HF), log(RSA), Log (RMSSD) and lower log(LF/HF) compared to non-breathing. All analyzed in FBS 2F+3F. Fetal heart rate was lower during breathing movements.
van Laar et al. (2014)	25/25	34–41	1,000	Active/rest	UL/FBS	Spectral	64 s epochs	RR-interval/ms	Total power, LF_absolute_ and LF_normalized_ were significantly higher in the active state compared to resting state. HF_absolute_ also increased but not significantly. HF_normalized_ was lower in the active state compared to resting state.
Brändle et al. (2015)	55/55	24–41	1,221	Active/rest	FMCG/FBS	Time domain Entropy	256 beats (moving window)	RR-interval/no units	Mean heart rate, RMSSD and (SDNN/RMSSD) increased with increasing activity from week 32. SDNN increased with increasing activity trough all gestational ages.
Arias-Ortega et al. (2016)	10/10	34.2 (± 2.6)	1,000	Breathing/non-breathing	UL	Time domain Spectral	30 s epochs	RR-interval/ms	Higher mean RR and RMSSD in fetal breathing compared to non-breathing.
Stone et al. (2017)	29/29	36–38	2,200	Active/rest	FBS	Time domain	1 min	RR-intervals/ms	SDNN and RMSSD were significantly higher in 2F compared with 1F and 4F. SDNN/RMSSD increased from 1F to 2F and from 2F to 4F. FHR was higher in 4F compared with 2F and in 2F compared with 1F.
Zavala et al. (2019)	22/22	36–39	300–900	Active/rest	FBS/AC	Time domain	30 s epochs	RR-intervals/ms	SDNN was significantly higher in 2F compared with 1F. In RMSSD a trend toward higher mean in 2F compared with 1F was observed (*p* = 0.09). No difference in FHR in state 2F and 1F.

*GA, Gestational age; UL, Real time doppler ultrasound; FBS, Fetal behavioral state defined by Nijhuis et al. (1982); dMMG, Magnetomyography; FMCG, Fetal magnetocardiography (changes in orientation of fetal heart vectors); AC, Fetal actocardiography*.

All studies found that time domain parameters, as well as spectral domain parameters, increased significantly during fetal movements compared to during fetal rest. Actually, most studies showed a two-fold or even higher increase during both fetal breathing movements and fetal body movements compared to fetal rest (Groome et al., 1994; Frank et al., 2006; Gustafson et al., 2011, 2012; Ortiz et al., 2013; van Laar et al., 2014; Brändle et al., 2015; Arias-Ortega et al., 2016). However, when drawing conclusions based on the details in this finding, it is necessary to discriminate between fetal breathing movements and fetal body movements, as different modes of fetal movements probably are associated with different parts of the autonomic response, thereby having different influences on heart rate variability.

### Fetal Breathing Movements

#### Time Domain Parameters and Fetal Breathing Movements

RMSSD, a recognized estimate of short-term parasympathetic activity, was the most studied time-domain parameter in the included studies. All studies report an increased RMSSD in breathing epochs compared to non-breathing epochs (Table 4) (Gustafson et al., 2011, 2012; Ortiz et al., 2013; Arias-Ortega et al., 2016). No clear difference in results between term and preterm pregnancies was seen, but gestational age in studies concerning term pregnancies only differed by 2 weeks from studies concerning preterm pregnancies.

**Table 4:** The association between RMSSD and fetal breathing activity, study overview and results.

**Study**		**Analyses**	**RMSSD**		
**References**	**Gestational age (weeks)**	**Specifications; unit**	**Breathing**	**Non-breathing**	***p*-value**
Gustafson et al. (2011)	36–38	RMSSD; ms^2^; median	8.2	5.6	0.006
Gustafson et al. (2012)^α^	>36	RMSSD; ms^2^; mean	6.36 (± 1.34)	4.85 (± 1.45)	<0.001
Ortiz et al. (2013)	34 (±3.7)	RMSSD; ms^2^; mean^β^	7.0	3.96	<0.05
Arias-Ortega et al. (2016)	34.2 (±2.6)	RMSSD; ms^2^; mean	8.2 (± 2.09)	4.8 (± 1.31)	<0.05

α *From Table 1 in Gustafson et al. (Gustafson et al., 2012), calculated by e^(logRMSSD)^*.

β *They write “conventional statistics of HRV: RMSSD”, but the unit is given as ms, but it must be ms^2^*.

SDNN, reflects all variance for a given period and includes both sympathetic and parasympathetic dependent HRV. However, SDNN estimates are very dependent on length, stationarity and mean of RR-intervals (mean RR) of the analyzed period. For timeseries <5 min in adults, SDNN reflects primarily parasympathetic activity (Heart rate variability, 1996). Wheeler et al. and Ohno et al. showed a higher SDNN during breathing compared to non-breathing epochs (Wheeler et al., 1980; Ohno et al., 1986). Ohno et al. supplied their analyses by the percentage of beat-to-beat differences below 2 milliseconds, which was lower during breathing compared to non-breathing, also indicating higher parasympathetic activity during breathing.

All results indicate a significant higher parasympathetic response measured by RMSSD and SDNN during fetal breathing compared to non-breathing.

#### Spectral Parameters and Fetal Breathing Movements

In term pregnancies, Ferrazzi et al. and Divon et al. reported a definite high frequency (HF) density-peak in the frequency range 0.7–0.9 Hz (Divon et al., 1985; Ferrazzi et al., 1989), which was validated in a larger cohort by Groome et al. (1994) (in adults the respiratory dependent peak is usually around 0.25 Hz corresponding to respiratory frequency of 15 per minute). Groome et al. also found a HF power peak within a relatively narrow frequency range (mean 0.63 ± 0.15 Hz, range 0.48–0.84 Hz), which correlates well to the vagal mediated respiratory-dependent sinus arrhythmia, corresponding to a respiratory frequency of 29 to 50 fetal breaths per minute. Additionally, a HF peak was present even in the absence of breathing activity although the mean amplitude of this peak was significantly lower than the average peak maximum observed during breathing (0.46 ± 0.53 vs. 1.63 ± 1.02, *p* = 0.001). This is in contrast to the findings of Ferrazzi et al. as they found no HF peak in non-breathing epochs, but according to the sample size and quality assessments studied by Groome et al. and Divon et al. appear more valid (Table 2).

In preterm fetuses (gestational age: 34 ±3.7), Ortiz et al. compared epochs dominated by fetal breathing movements with epochs dominated by non-breathing (incidence of fetal breathing movements in the two groups: 64.9 vs. 13.3%; *p* < 0.0001). As in term pregnancies, an increase in HF power was found in epochs dominated by fetal breathing movements compared to epochs dominated by non-breathing (Ortiz et al., 2013). Arias-Ortega et al. confirmed the finding of increased HF power during breathing in preterm fetuses (Arias-Ortega et al., 2016). However, neither LF power, LF/HF ratio nor normalized HF power, and thereby no proportional HF change, is shown in either of the two studies. Only Ferrazzi et al. included fetuses below 30 weeks of gestation. They found no HF power in fetuses at 26 weeks of gestation neither during breathing movements nor in non-breathing periods. However, these results have low evidence due to very few participants and poor description of data processing (Table 2).

#### Fetal Breathing Movements and the Sympatho-Vagal Balance

Only Groome et al. and Gustafson et al. presented both LF (an estimate of primarily sympathetic activity but also includes some parasympathetic activity) and HF power (Groome et al., 1994; Gustafson et al., 2011, 2012). The two works by Gustafson et al., found a significant decrease in LF/HF ratio in breathing epochs compared to non-breathing, while Groome et al. describe no change in the distribution of total power in the different frequency bands in breathing epochs compared to non-breathing (Table 5).

**Table 5:** The association between spectral indexes and fetal breathing activity, study overview and results.

**Study**			**Frequency bands**		**Analyses**	**LFabsolute**			**HFabsolute**			**LF/HF**		
**References**	**GA^a^**	**FBS^b^**	**LF (Hz)**	**HF (Hz)**	**Specifications; unit**	**Breathing**	**Non-breathing**	***p*-value**	**Breathing**	**Non-breathing**	***p*-value**	**Breathing**	**Non-breathing**	***p*-value**
Groome et al. (1994)^c^	37.9 (±1.1)	F1	0.04–0.2	0.2–2.5	Power density; no units given	0.62	0.33	<0.05	0.77	0.42	0.001	0.81^e^	0.79^e^	
Gustafson et al. (2011)^d^	36-38	F2+F4	0.08–0.2	0.4–1.7	Power density; bpm^2^	1.42	1.39	0.81	1.31	0.63	<0.001	1.08	2.21	0.004
Gustafson et al. (2012)^c^	>36	F2+F4	0.08–0.2	0.4–1.7	power density; ms^2f^	10.1	12.4	0.74	9.1	4.3	<0.001	1.1	3.2	<0.0001
Ortiz et al. (2013)^cg^	34 (±3.7)	None		0.3–2.0	Power density; ms^2^				15.6?	7.8	<0.001			
Arias-Ortega et al. (2016)^c^	34.2 (±2.6)	None		0.5–1.5	AMP_HF;_ ms^2^				4.47	2.24	<0.05			

a *Gestational age*.

b *Fetal behavioral state(39). 1F: quiet, brief gross body movements, mostly startles; 2F: active, frequent and periodic gross body movements; 4F: active, vigorous, continual activity including many trunk rotations*.

c *Mean*.

d *Median*.

e *Calculated according to results given in the study*.

f *Calculated by exp[log(power)]*.

g *Breathing: ≥ 40% breathing movements, non-breathing: <40% breathing movements*.

#### FHRV and Fetal Breathing Movements in Specific Fetal Behavioral States (FBS)

Three of the included studies concerning fetal breathing movements in term pregnancies restricted their analyses to specific fetal behavioral states (FBS) as defined by Nijhuis et al. (1982).

Only epochs of fetal heart rate classified as fetal behavioral state 1F (quiet) were included by Groome et al. Nevertheless, they found a significant difference in total power, Very Low Frequency power (VLF), LF, HF and a frequency component corresponding to RSA (frequency-band: 0.4–1.0 Hz) when comparing epochs of fetal breathing movements with non-breathing (Groome et al., 1994). Gustafson et al. included epochs classified as 2F and 4F (active), and they also found a significant difference in HF but not LF and total power when comparing epochs of breathing and non-breathing (Gustafson et al., 2011, 2012). In addition, they performed a time domain analysis including RMSSD, which was significantly higher in fetal breathing compared to non-breathing epochs in the 2F and 4F states.

#### FHRV and RR-Intervals

Gustafson et al. found a significant difference in heart rate (HR), with a lower mean HR during breathing, which is in accordance with a higher mean RR found by Arias-ortega during breathing. In contrast, Wheeler et al. Ohno et al. and Ortiz et al. found no difference in mean RR between the two groups (Wheeler et al., 1980; Ohno et al., 1986; Ortiz et al., 2013). However, Ortiz et al. also studied the association between FHRV parameters and fetal RR-interval and found that both RMSSD and HF power showed a significant linear correlation with RR-intervals in non-breathing epochs, but not in breathing epochs. RMSSD and HF power increased with increasing RR interval but only in the non-breathing group.

### Fetal Body Movements

Six (Vergales et al., 2014) of the included studies present FHRV indexes in relation to fetal body movements or active FBS (2F and 4F) compared to rest/resting state (1F) as defined by Nijhuis et al. (1982).

#### Time Domain Parameters and Fetal Body Movements

Especially SDNN is well-studied. Wheeler et al. showed a significant association between active fetuses and SDNN with a higher SDNN in active fetuses compared to resting fetuses in term pregnancies (Wheeler et al., 1980). This was confirmed by Frank et al. (2006), Brändle et al. (2015) Stone et al. (2017), and Zavala et al. (2019). An increase in RMSSD was also found. Nevertheless, proportionally SDNN increased more than RMSSD during fetal activity (Brändle et al., 2015; Stone et al., 2017; Zavala et al., 2019).

Brändle et al. included pregnancies from 24 weeks of gestation and reported an increasing SDNN/RMSSD ratio with increasing fetal activity from gestational week 32. SDNN increased with increasing activity through all gestational ages.

#### Spectral Parameters and Fetal Body Movements

In a cohort primarily consisting of term pregnancies, Van Laar et al. found that both LF_absolute_ and LF_normalized_ were higher in the active state compared to the quiet state. HF_absolute_ also showed a tendency toward an increase in active compared to resting state, but it was not significant, and HF_normalized_ actually decreased in the active state compared to the resting state (van Laar et al., 2014). Frank et al. found that both ln(LH) and ln(HF) were able to discriminate between state 1F from 2F or 4F, and from a boxplot it is shown that both parameters are lower in 1F compared to 2F and 4F. Unfortunately, no absolute values of HRV parameters are given (Frank et al., 2006).

#### Entropy Parameters and Fetal Body Movements

Only one of the studies analyzed approximate entropy (ApEn_sub_, ApEn_pop_), where ApEn_sub_ showed a decrease from F1 (quiet) to F4 (active, awake) and ApEn_pop_ increased from F1 (quiet) to F2 (active, sleep), however no difference between F1 and F4 was seen in ApEn_pop_ (Frank et al., 2006). Hence, no clear tendency in ApEn was seen when comparing the quiet state (F1) to the active states (F2, F4).

#### Fetal Body Movements and the Sympatho-Vagal Balance

All studies agree on higher variability in active fetuses, but also a tendency toward a higher sympatho-vagal ratio as LF_normalized_ increased, HF_normalized_ decreased and SDNN/RMSSD seems to be higher in the active fetus compared to the resting fetus. Additionally, Stone et al. found FBS 4F to have the highest SDNN/RMSSD (Stone et al., 2017), indicating that the sleeping fetus [both moving (2F) and not moving (1F)] had a proportionally higher parasympathetic response than the awake and active fetus (4F). However, all studies used short time series of up to 5 min duration, where SDNN primarily represents parasympathetic activity, but also sympathetic activity is reflected. Additionally, the validity of SDNN/RMSSD as a measure of sympatho-vagal balance in very short timeseries is not well-established.

## Discussion

### Main Findings

The most important finding of this review was that time domain as well as spectral domain parameters are affected by fetal movements. All included studies showed an increase, and most studies showed a two-fold increase in these analyses in the moving fetus compared to the resting fetus.

During periods with fetal breathing movements, indexes of especially parasympathetic activity increased (higher RMSSD and HF power, lower LH/HF power) even within a certain FBS. Fetal body movements were associated with a higher SDNN, SDNN/RMSSD and LF_normalized_ and lower HF_normalized_, which can be related to sympathetic dependent shifts in mean RR level, but also other changes in autonomic activity.

### Strengths and Limitations

Rather strict inclusion criteria reduced the risk of bias. Only healthy fetuses and healthy pregnant women were included and important selection bias were thereby avoided (Bekedam et al., 1985; Vindla et al., 1999; Yeoshoua et al., 2012; Arias-Ortega et al., 2016; Fehlert et al., 2017). Furthermore, only studies concerning pregnancies prior to labor were included to avoid the possible influence from contraction on FHRV. Additionally, fetal breathing movements are almost abolished during labor (Richardson et al., 1979). It is well-known that a low sampling frequency has an impact on time domain and spectral domain analyses, especially the HF power. By restricting the analyses based on FECG and FMCG, some of this effect was prevented, but it is a limitation that results from a few of the included studies are based on a low sampling frequency. However, presuming that the effect of a low sampling frequency is non-differential between compared groups, this is not a major source of information bias. The findings of this review would have probably been even clearer if studies with higher sampling frequency and higher quality of data processing had been available.

An association between time domain and spectral domain indexes and gestational age, with increasing variability (SDNN, RMSSD, HF, LF) through 2nd and 3rd trimester, is widely accepted and well-documented (Van Leeuwen et al., 2003, 2013; Hoyer et al., 2009; van Laar et al., 2014). All included studies specified gestational age and restricted their inclusion of participants to few gestational weeks or divided their results into specific gestational age groups, which is very important when validating the findings of this review.

Based on the rate of fetal breathing movements, the HF frequency band should be altered from the recommended intervals in adults (Heart rate variability, 1996), to include the frequency of breathing movements in fetuses. All studies covered the HF frequency band 0.5 to 1.5 Hz corresponding to a frequency of 30–90 breaths per minute, which is appropriate in fetuses (Dornan et al., 1984) and therefore also a strength when comparing results between studies.

The strongest risk of bias is introduced by the quality of the algorithm for R-wave detection and the elimination of RR intervals due to noise and artifacts. Many studies do not describe how the algorithm was performed and how much interpolation for missing RR-intervals was allowed. The signal quality of the FECG might depend on fetal movements and thereby influence detection rate of R-waves, which may lead to a risk of non-differential misclassification.

Another possible source of information bias is that only a few studies have reported how they have selected the time series used for analysis. This is very important as large trends in mean RR (non-stationarity) have an impact on results. By focusing on specific FBS, some studies coped with possible bias due to non-stationarity especially Groome et al. who used the F1 state, which prevents large fluctuations in RR-intervals in analyzed time series. The length of time period used for analysis may also impact results. The shorter the time period, the more the results are dominated by parasympathetic variability, so time periods should principally be standardized to allow for meaningfully comparability. The majority of studies analyzed time periods of <5 min, with most around 1–2 min in both groups. This is especially so in spectral analyses, which most studies report are rather sensitive to length of time periods and noise, whereas time-domain analyses are more resistant to length of time period and noise.

Quantitative analyses including meta-analysis were not relevant due to the heterogeneity in the sampling procedures, calculation methodology and small sample size; in addition, reporting was lacking on results including missing confidence intervals, standard deviations and even units in the earliest studies. However, assumptions on more general tendencies and associations can be drawn from the current data.

### Interpretation

The findings of this review indicate an increase in time domain and spectral domain parameters during fetal movements, most evidence relating to fetal breathing movements. Especially short-term, vagal mediated indexes are shown to be increased during fetal breathing movements. A significantly lower LF/HF ratio during breathing compared to non-breathing is expected, as HF power is primarily known to represent the parasympathetic response and contains oscillations in heart rate associated with RSA (Katona and Jih, 1975; Eckberg, 1983). Principally, an increase in vagal tone results in an increase in the RR-interval, which is in line with some studies that find a lower HR (higher mean RR) in fetuses during breathing movements (Gustafson et al., 2011, 2012; Arias-Ortega et al., 2016); however, other studies report no difference in mean RR (Wheeler et al., 1980; Ohno et al., 1986; Ortiz et al., 2013). However, the non-linear relationship between heart rate and RR-interval may bias variability analyses and explain some of the association between fetal breathing movements and heart rate variability, as none of the included studies adjusted their results for average RR-interval.

FBS is often used to determine fetal activity in studies on FHRV. However, Groome et al. and Gustafson et al. found the same significant associations between time domain and spectral domain analysis and fetal breathing movements even though they only included 1F (resting fetus, Groome) and 2F, 3F (active fetus, Gustafson). Fetal breathing movements are inconsistent, also in the specific FBS (Pillai and James, 1990b). FBS is based on the evaluation of gross movements, eye movements and heart rate pattern, not breathing movements, and fetal breathing movements are common but not constant in FBS. This fact should be considered in studies where fetal activity is determined by FBS and FHRV is interpreted.

Only one study reporting on spectral analyses included pregnancies prior to 30 weeks of gestation and found no density peak corresponding to RSA during fetal breathing movements. However, other studies based on fetal HRP describe an association between “active” HRP and both time domain and spectral domain analyses even in very preterm fetuses (Hoyer et al., 2009). David et al. found a HF peak in very preterm fetuses, although weaker than in preterm and term fetuses (David et al., 2007). However, it is essential to discriminate between an active fetal HRP and an active fetus when assessing the effect of fetal movements, as a reactive fetal HRP not necessarily correlate to fetal movements in these early gestational ages.

From this study, there is no conclusion on whether fetal HRP or FBS is usable in the process of selecting epochs appropriate for FHRV analyses. Although standardization and interpretation of the results of both time domain and spectral domain analyses seem to be improved, when adding fetal movements, including fetal breathing movements, into the process of selecting timeseries for heart rate variability analyses.

Leaving fetal movement out of the FHRV assessment may lead to loss of important information. In the aspect of fetal neurophysiology important knowledge clarifying the development of fetal sinus arrythmia and the fetal autonomic nervous system might be omitted.

In the clinical setting, leaving fetal movements out may lead to incorrect assessment of FHRV, which in the worst case could give rise to missing identification of a compromised fetus.

Recommendations in clinical and scientific use of FHRV are needed. However, high-quality transparent studies needs to reveal the reliability of FHRV, including the impact of fetal movements, gestational age and methodological factors as length of time series and correction of R-waves on FHRV. This should be included in studies of intra- and inter-observer reliability.

## Conclusion

Fetal breathing movements were associated with an increased FHRV, primarily in parameters supposed to represent parasympathetic activity, even when restricting them to a specific FBS. On the other hand, parameters which can be related to sympathetic activity increased during fetal body movements. Fetal movements should be considered in FHRV, in the scientific aspect, to achieve a more detailed interpretation of results and, in the clinical aspect, to minimize the risk of misinterpreting results of FHRV.

## Data Availability Statement

The original contributions generated for the study are included in the article/supplementary material, further inquiries can be directed to the corresponding author/s.

## Author Contributions

AZ did the literature search together with a librarian (Cand.Scient.Bibl.). AZ and IK screened titles and abstracts, crosschecked reference lists for further relevant papers, and conducted a full-text screening based on the eligibility criteria. AZ and NU conducted the quality assessment. All authors contributed to the data extraction, writing process, revised the paper critically for important intellectual content, made substantial contributions to the conception and design of the article, and gave final approval of the version to be submitted.

## Conflict of Interest

The authors declare that the research was conducted in the absence of any commercial or financial relationships that could be construed as a potential conflict of interest.

## Abbreviations

FHRV: Fetal heart rate variability
CTG: Cardiotocography
FMCG: Fetal magnetocardiography
FECG: Fetal electrocardiography
NI-FECG: Non-invasive fetal electrocardiography
RSA: Respiratory sinus arrhythmia
RMSSD: Root Mean Square of the Successive Differences
NN: Intervals between adjacent QRS complexes from normal sinus beats
Mean RR: Mean interval between adjacent QRS-complexes in milliseconds
SDNN: Standard Deviation of NN
HF: High Frequency power
LF: Low Frequency Power
VLF: Very Low Frequency power.
