# Supplementary material for: Fetal Heart Rate Variability Is Affected by Fetal Movements: A Systematic Review
Source: Front Physiol. 2020 Sep 30;11:578898. doi: 10.3389/fphys.2020.578898 (PMC7554531; doi:10.3389/fphys.2020.578898)
Supplement: Supplementary file 1 [file Table_1.docx]

Appendix 1: Research PICO-question

Is fetal heart rate variability associated to fetal movements in uncomplicated singleton pregnancies?

***Population:*** Singleton uncomplicated pregnancies.

***Exposure:*** Fetal movements including fetal gross movements and fetal respiratory movements.

***Comparator:*** Fetuses not moving.

***Outcome:***  Fetal heart rate variability. Primary outcomes: Time domain and spectral domain analyses. Secondary outcome: Entropy analyses

***Study design:*** All studies
